# The role of delayed bone age in the evaluation of stature and bone health in glucocorticoid treated patients with Duchenne muscular dystrophy

**DOI:** 10.1186/s13633-019-0070-0

**Published:** 2019-12-23

**Authors:** E. J. Annexstad, J. Bollerslev, J. Westvik, A. G. Myhre, K. Godang, I. Holm, M. Rasmussen

**Affiliations:** 1 Department of Neurology, Unit for Congenital and Inherited Neuromuscular Disorders, Oslo University Hospital, PoBox 4950, Nydalen, 0424 Oslo, Norway; 2 Faculty of Medicine, University of Oslo, Oslo, Norway; 3 Department of Clinical Neurosciences for Children, Oslo University Hospital, Oslo, Norway; 4 Department of Endocrinology, Section of Specialized Endocrinology, Oslo University Hospital Rikshospitalet, Oslo, Norway; 5 Department of Radiology, Section for Paediatric Radiology, Oslo University Hospital Rikshospitalet, Oslo, Norway; 6 Frambu Resource Centre for Rare Disorders, Siggerud, Norway; 7 Division of Orthopaedic Surgery, Oslo University Hospital, Oslo, Norway; 8 Children’s Department, Ostfold Hospital Trust, Sarpsborg, Norway

**Keywords:** Duchenne muscular dystrophy, Glucocorticoid, Bone age, Bone mineral density, Pubertal delay, Short stature

## Abstract

**Background:**

Low bone mineral density and an increased risk of appendicular and vertebral fractures are well-established consequences of Duchenne muscular dystrophy (DMD) and the risk of fractures is exacerbated by long-term glucocorticoid treatment. Monitoring of endocrine and skeletal health and timely intervention in at-risk patients is important in the management of children with DMD.

**Methods:**

As part of the Norwegian Duchenne muscular dystrophy cohort study, we examined the skeletal maturation of 62 boys less than 18 years old, both currently glucocorticoid treated (*n* = 44), previously treated (*n* = 6) and naïve (*n* = 12). The relationship between bone age, height and bone mineral density (BMD) Z-scores was explored.

**Results:**

The participants in the glucocorticoid treated group were short in stature and puberty was delayed. Bone age was significantly delayed, and the delay increased with age and duration of treatment. The difference in height between glucocorticoid treated and naïve boys was no longer significant when height was corrected for delayed skeletal maturation. Mean BMD Z-scores fell below − 2 before 12 years of age in the glucocorticoid treated group, with scores significantly correlated with age, duration of treatment and pubertal development. When BMD Z-scores were corrected for by retarded bone age, the increase in BMD Z-scores was significant for all age groups.

**Conclusion:**

Our results suggest that skeletal maturation should be assessed in the evaluation of short stature and bone health in GC treated boys with DMD, as failing to consider delayed bone age leads to underestimation of BMD Z-scores and potentially overestimation of fracture risk.

## Background

The physiological process of bone modelling and remodelling in childhood involves complex and sensitive endocrine and immunological signalling systems, which may be affected by factors such as physical conditioning, poor linear growth, obesity, pubertal delay, vitamin D deficiency, and alterations in calcium homeostasis [1, 2]. Low Bone Mineral Density (BMD) and an increased risk of fractures is a well-established consequence of many chronic diseases in childhood, including Duchenne muscular dystrophy (DMD) [3, 4].

Several studies have described a higher incidence of low-trauma long-bone (appendicular) fractures in steroid-naïve boys with DMD compared to the normal population [5, 6], and a significantly increased risk of appendicular and vertebral fractures in glucocorticoid (GC) treated DMD [7].

In untreated DMD, it is suggested that reduced bone strength is related to decreased muscle tension on bone and muscle disuse as well as nutritional factors, disturbances in the calcium homeostasis and increased activity of inflammatory cytokines [1, 8]. Histomorphometric analyses of bone biopsies in DMD showed clear differences in all compartments between patients with DMD compared to healthy age-matched reference data. Thus, the total length of the bone biopsy was reduced, as was the cortical shell (thickness). Moreover, the total trabecular bone volume per total volume was reduced, due to a reduction in trabecular thickness. These findings indicate significant reduction in bone formation and a relatively increased bone resorption (increased endosteal resorption) [9]. GC treatment exacerbates the situation by disrupting the osteoblast-osteoclast balance and coupling [10], compromising the biomechanical properties of bone [11], and inhibiting the absorption of calcium from the gastrointestinal tract and the reabsorption of calcium in the renal tubuli [10]. GC treatment is also associated with 25-hydroxy-vitamin D (25(OH)D) deficiency and by itself increased fracture risk [12]. Further, steroid induced obesity, poor linear growth [13], hypogonadism and pubertal delay [14] add to the complexity of bone health evaluation in GC treated DMD [15] .

Fractures in the long-bones are painful and can be detrimental to the ambulatory function of a patient with DMD, especially if they occur in the late-ambulatory phase [5]. Vertebral fractures may cause severe pain, but may also be asymptomatic and thereby underdiagnosed [16]. The long-term consequences of permanent deformity on pain and function following repeated childhood vertebral compression remain unstudied [4]. Patients with mild or even asymptomatic fractures may have a high risk of future vertebral fractures [16, 17]. Bone health monitoring, identification of at-risk patients and timely intervention is thus an important part of the management of DMD.

Dual-energy X-ray absorptiometry (DXA) is the standard measure of bone mass and density for clinical use in children [18, 19]. However, DXA has important limitations in the evaluation of the fracture risk in a growing child. Bone strength is also dependent on bone size, geometry, architecture, bone matrix quality, and anthropometric variables, which may be independent of BMD [18, 19]. Children can have microarchitectural changes and an increased fracture risk even with a normal BMD [4, 20].

In children with short stature or growth delay, BMD is systematically underestimated due to the two-dimensional presentation of bone area (g/cm2) in which bone thickness or depth is not factored into DXA estimates of BMD [21]. The International Society for Clinical Densitometry (ISCD) recommend that bone mineral content (BMC) and BMD in this situation should be adjusted using either bone mineral apparent density (BMAD) [22] or height-for-age Z-score (HAZ) [23] for the spine, and HAZ for total body less head measurements (TBLH) [19]. However, no prediction equation for bone mass based on height, weight and bone area has been published [20].

As a part of the Norwegian Duchenne muscular dystrophy cohort study [24], we have previously reported preliminary findings of considerably retarded bone maturation in a group of GC treated DMD patients [25]. We have also reported that the delay in skeletal maturation increases with age [25]. Bone age in adolescence is closely related to pubertal development and growth spurt, which are stunted in GC treated DMD [14, 26]. Therefore, our aim was to examine the extent of delayed bone age in boys with GC treated DMD, and to explore the implications of such delay for the evaluation of short stature and low BMD Z-scores. We hypothesized that a delay in bone age is closely related to the stunted growth and low BMD Z-scores in GC treated DMD. Furthermore, that taking bone age into account in the evaluation of BMD in GC treated boys with DMD is imperative, as the delay may influence evaluations of bone health in this patient group.

## Methods

### The Norwegian Duchenne muscular dystrophy cohort study

Sixty-five of 94 (69%) identified male cases of clinically, biochemically (Creatine Kinase, CK) and genetically confirmed DMD aged 0–18 years were recruited to the study over a period of 3.5 years. Recruitment was based on written consent following initial contact with the boys and/or their legal guardian from their local Child rehabilitation clinic. Twenty-nine (31%) individuals declined clinical participation or were diagnosed too late for recruitment. Participants included boys aged 2–18 years, with a mean age of 11.0 years (SD ± 4.0 years). Participants were both GC treated (*n* = 45), previously GC treated (*n* = 6) and GC naïve (*n* = 14).

The study was approved by The Regional Ethics Committee and the Data Protection Officer at Oslo University Hospital, and registered with ClinicalTrials.gov, number NCT01963897.

Participants met with the first author (EJA) annually on one, two or three occasions depending on their time of recruitment. All participants were subjected to extensive clinical, biochemical and radiological examinations. The participants and their parents or guardians were interviewed, and the boys’ medical records were collected for review in order to ensure correct retrospective information.

The results presented in this article are part of the larger cohort study [24], with focus on growth, skeletal maturation and bone health. The relevant parts of the study protocol are described below.

### Demographic data

The participants’ height and weight were measured. When the participant was unable to stand or lay flat, height was measured by arm span (middle finger to middle finger) [27, 28]. Percentiles and iso-BMI (body mass index) were calculated according to the Centers for Disease Control and Prevention (CDC) reference data [29]. Pubertal development (gonads and pubic hair combined) was indicated by self-assessment [30] according to the Tanner scale [31]. North Star Ambulatory Assessment [32] was performed for ambulant boys over 5 years of age. For non-ambulatory boys, a modified Vignos [33] & Brooke [34] motor function assessment was used. Clinical examinations, laboratory investigations as well as radiographs of the left hand and wrist and DXA were performed at the same time points.

Historical data were collected from the participants’ medical records, including time of diagnosis, GC treatment initiation, growth, independent ambulation and loss of ambulation if relevant. All long bone and vertebral fractures including type of trauma, age and GC status at the time of fracture were noted.

### Glucocorticoid treatment

Prednisolone is the first drug of choice for DMD of the Norwegian medicinal regulatory authorities, and the majority of GC treated boys with DMD are first prescribed daily Prednisolone. Deflazacort is available for patients who experience intolerable side-effects of Prednisolone. In accordance with consensus guidelines, the recommended starting doses are 0.75 mg/kg/d for prednisolone and 0.9 mg/kg/d for deflazacort, both equivalents to hydrocortisone 3 mg/kg/d [35, 36]. In clinical practice, the balance between benefits and side-effects often results in lower doses per kg bodyweight prescribed for older patients [24].

At the time of our final inquiry, 19/65 boys (29%) were on daily Prednisolone, while 26/65 (40%) were prescribed deflazacort. Mean GC doses are summarised in Table 1. Six boys (9%) had stopped GC treatment due to unacceptable side-effects, of which excessive weight-gain and pubertal delay were the most common. Fourteen boys (22%) had never received GC treatment [24].

**Table 1:** Key characteristics of the participants age, growth, weight, pubertal development and glucocorticoid (GC) treatment

	Prednisolone	Deflazacort	GC previously	GC naïve	Total
	*n* = 19	*n* = 25	*n* = 6	*n* = 12	*n* = 62
Age	9.6 (±3.0)	11.6 (±2.9)	16.6 (±1.4)	8.9 (±5.8)	11.0 (±4.2)
Age at diagnosis	4.3 (±1.8)	3.6 (±1.9)	5.3 (±2.7)	3.8 (±2.3)	4.0 (±2.0)
Age at loss of ambulation	10.5 (±1.8) *n* = 3 (15.8%)	10.8 (±2.5) *n* = 6 (25.0%)	11.6 (±1.9) *n* = 6 (85.7%)	9.6 (±0.9) *n* = 5 (41.7%)	10.7 (±1.9) *n* = 20 (32.3%)
GC initiated	6.1 (±2.3)	5.3 (±1.3)	7.8 (±1.6)	N/A	5.9 (±1.9)
GC duration	3.8 (±3.2)	6.3 (±3.3)	4.6 (±3.0)	N/A	5.2 (±3.4)
GC stopped	N/A	N/A	11.9 (±3.1)	N/A	11.8 (±3.1)
GC dose at time of investigation^a^	0.49 (±0.13) mg/kg/d	0.49 (±0.20) mg/kg/d	N/A	N/A	N/A
Height for age perc. (95% CI)	13.0 (4.4, 21.7)	5.2 (0.2, 10.2)	28.0 (2.2, 53.7)	32.2 (14.4, 49.9)	15.5 (10.0, 21.1)
Weight for age perc. (95% CI)	62.2 (46.3, 78.0)	41.8 (27.6, 56.1)	52.1 (8.4, 95.9)	54.3 (34.7, 73.9)	51.6 (42.8, 60.3)
Iso-BMI	21.6 (4.7)	22.4 (5.2)	22.1 (8.6)	18.3 (2.6)	21.2 (5.0)
Tanner	1–2	1–3	2–5	1–5	

Age and timing of milestones in years ±SD, unless otherwise indicated. Height and weight are indicated as CDC percentiles. *CI* Confidence interval. *N/A* not applicable. ^a^Hydrocortisone equivalent dose 1.96 mg/kg/d for prednisolone and 1.63 mg/kg/d for deflazacort.

### Laboratory investigations

Extensive laboratory investigations were performed in the morning (before 10 am) of hospital visits at one-year intervals, including biochemical markers of bone metabolism and endocrine function tests. FSH and LH was measured by non-competitive immunometric assay (Simens, Immulite XPI 2000) at the Hormone Laboratory, Oslo University Hospital, Norway, and testosterone on an LC-MS/MS method developed at the Hormone Laboratory, Oslo University Hospital [37]. The reference intervals for LH and FSH for prepubertal (Tanner stage 1) boys are established by the manufacturer at LH < 0.1–0.8 IU/L and FSH 0.2–2.1 IU/L. For testosterone, paediatric reference intervals are taken from Kushnir et al. (Tanner stage 1 ≤ 0.59 nmol/l [38].

### Bone age assessment

Sixty-two participants, both GC treated and GC naïve, were subjected to radiological examination of the left hand and wrist on one, two or three occasions. Three participants missed their radiology appointments for personal reasons. Radiographs were taken according to standard bone age procedure at various hospitals throughout the country, and transferred digitally to Oslo University Hospital for evaluation by a single, expert paediatric radiologist (JW).

Skeletal maturation was evaluated according to the Greulich and Pyle (G&P) atlas, comparing the size and maturation of the epiphyses of the left hand and wrist to images reflecting statistically defined stages of normal skeletal development and growth throughout childhood and adolescence [39]. The G&P atlas specifies mean ages of attaining consecutive developmental stages, including limits of normal variation (standard deviation, SD). Deviation of more than 2SD in either direction indicates severe maturation delay or advancement respectively.

In the present study, bone age was indicated at 6 months’ intervals (e.g. 12.0, 12.5 years) based on the mean age of attaining the corresponding developmental stage according to the G&P atlas. Where participants had been subjected to repeated measures, the latest obtained radiograph for each participant was chosen for statistical analysis.

**Fig. 1:**
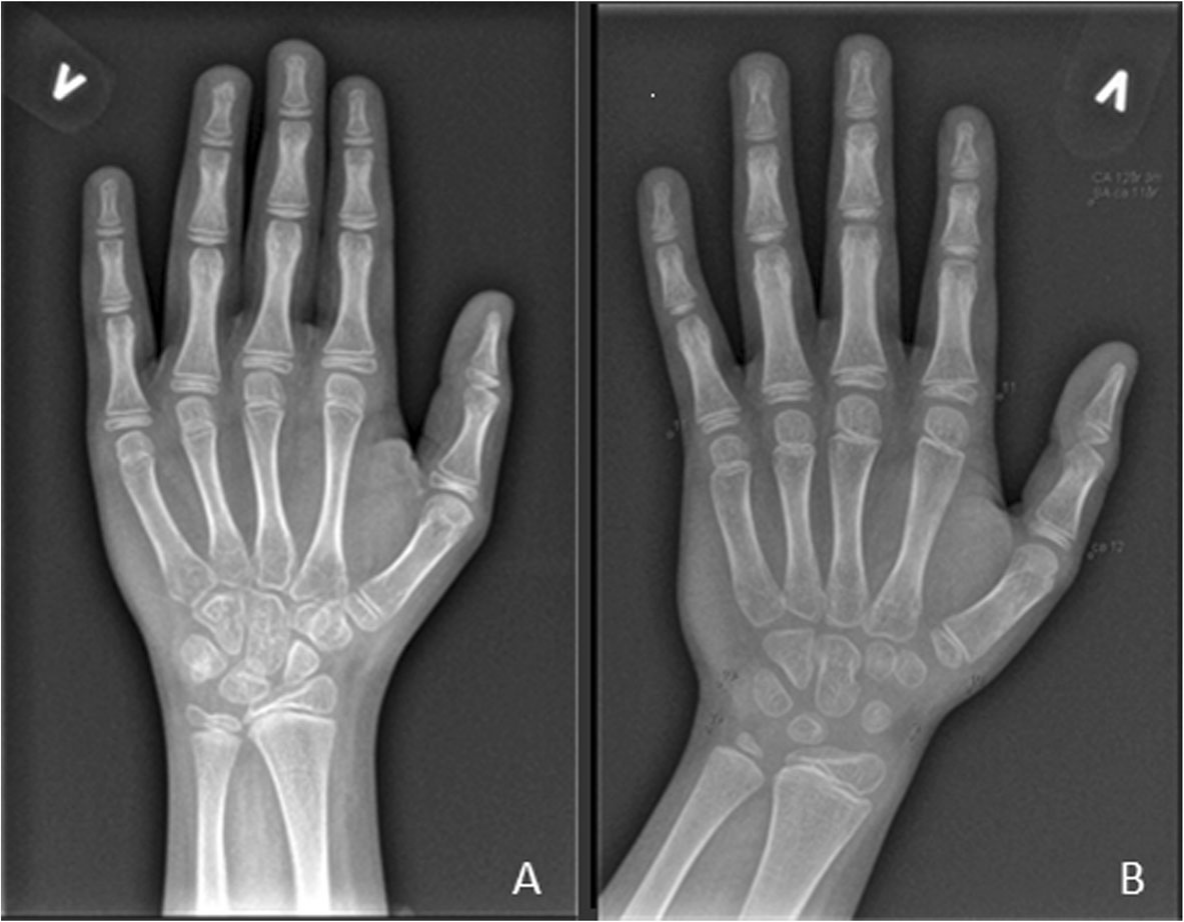
**a**: CA 12 y 5 m. GS naïve. BA 12 y 6 m in the carpals and the phalanges. **b**: CA 12 y 3 m. GC from 7 y. Distal ulna 7 y, scaphoid 9 y, phalanges 11y 6 m. CA: Chronological age. GC: Glucocorticoid. BA: Bone age. y: years. m: months

As previously described [25], in many cases there was a marked difference in skeletal maturation between the carpals and the phalanges. In some boys maturation of the carpals were retarded several years compared to the phalanges (Fig. 1). The clinical relevance of these findings is yet unclear. For the analyses, the bone age of the phalanges, which was closer to the chronological age, was recorded and used in statistical analyses.

### DXA measurement

Thirty-nine of 65 participants were examined with DXA on one, two or three occasions, at Oslo University Hospital Rikshospitalet (*n* = 27), the University Hospital of North Norway, Tromso (*n* = 7) or Haukeland University Hospital, Bergen (*n* = 5). Seventeen participants were either unable to position on the DXA table, too young for reference data (< 5 years of age), or missed their appointment. Measurements from 9 of the 65 participants were excluded from analysis due to metal implants from scoliosis surgery, fracture fixation or other technical difficulties. Where participants had been subjected to repeated measures, the latest obtained DXA for each participant was chosen for statistical analysis.

BMD was measured with DXA at the anterior - posterior lumbar (L2 - L4) spine and total body less head (TBLH), in accordance with the ISCD official position [19]. The BMD measurements were performed using a narrow fan beam DXA densitometer (GE Healthcare, Madison, WI, USA) in Oslo, Lunar Prodigy in Tromso or Lunar iDXA in Bergen. No hardware changes were made during the study period. All the scans were analysed or re-analysed in the same software version 16 [SP2] (same manufactory).

All three DXA centres had procedures for calibrations for BMD to avoid systematic errors between different DXA scanners. The DXA scanning procedure has been described in detail elsewhere [40].

BMD Z-scores were estimated by comparison to the Lunar reference database incorporated in the software. The database includes BMD data from healthy subjects from the general American population. Absolute BMD values are expressed as grams of mineral per square centimetre (g/cm^2^) and Z-scores were estimated by comparison to the reference population, which has been validated as suitable for clinical use in the adult Norwegian population [41]. Based on the adult validation study, suitability for the paediatric population was inferred.

Of the original 39 boys subjected to DXA, scans from 34 boys (87.2%) were re-analysed by an ISCD Certified Densitometry Technologist (KG) based on corrected bone age. Measurements from 5 boys were excluded from bone age analyses due to practical difficulties in importing raw data from other hospitals.

The remaining 34 boys had their DXA performed at Oslo University Hospital or the University Hospital of North Norway. As the GE software only indicates a bone age- adjusted BMD Z-score graphically, but does not provide the actual Z-score in numbers, KG adjusted all birth dates in accordance with the given bone age.

### Statistical analyses

Data collection and clinical examinations were performed by the first author. All personal data were replaced with consecutive case numbers by a research assistant. All data were then entered into a database by the first author, and analysed using the IBM SPSS Version 23 software.

Demographic and clinical data were subjected to descriptive analyses. Normality tests were performed to avoid violation of required assumptions of normal distribution, linear relationships and sufficient sample size for parametric tests. Paired-samples t-tests were performed in order to compare matched values of chronological age and bone age within the same group and independent samples t-tests compared mean values between the two GC subgroups (prednisolone vs deflazacort). One-way between groups analyses of variance (ANOVA) including post-hoc comparisons were used to compare height percentile scores between multiple groups. A bivariate Pearson correlation coefficient was obtained for correlation analyses.

## Results

### Demographic data

Sixty-two of the original 65 participants were included in the bone age study. Three participants were excluded due to missing data. There was no indication of growth hormone deficiency in any participant. Key characteristics of the participants are summarised in Table 1**.**

Thirty-one of 59 boys (52.5%) for whom height measurements were available had a height below the 5th CDC percentile, and thus met the definition of short stature. Among the boys with short stature were 1 of 6 previously GC treated (16.7%), 2 of 11 GC naïve (18.2%), 10 of 19 on treatment with Prednisolone (52.6%) and 18 of 23 on treatment with deflazacort (78.3%).

Between-groups ANOVA revealed a significant difference in height percentiles between the GC naïve group and both GC treated subgroups. Apparent differences in weight percentiles between the subgroups were not significant.

Visual inspection of CDC growth charts of the GC treated participants, based on historic data from their medical records, revealed considerable and increasing arrest of linear growth following the initiation of GC treatment. The linear growth of the GC naïve boys remained within the same percentile throughout childhood.

CDC growth charts of the 6 participants who had discontinued GC treatment, shortly before or after loss of ambulation, revealed a marked catch-up growth in height and pubertal development following the cessation of GC treatment.

Pubertal delay was common in the GC treated group. Nine GC treated participants aged 13.8–16.2 years reported having reached Tanner stage 2 without systemic puberty induction treatment, but biochemical indications of pubertal development was only found in two boys aged 14.4 and 15.7 years (morning testosterone 0.4 nmol/l, LH 1.7 IU/L and FSH 2.2 IU/L, and morning testosterone 2.3 nmol/l, LH 6.9 IU/L and FSH 8.4 IU/L, respectively). Two GC treated boys had received systemic testosterone treatment. At age 15, their pubertal development corresponded to Tanner stage 2 and 3. In the remaining 40 GC treated boys, including 5 boys over 14 years of age, no biochemical evidence of puberty was found. In contrast, the GC naïve boys progressed through normal pubertal development.

### Bone age

The difference between chronological age (CA) and bone age (BA) for the whole study group covered a wide range from bone age advancement of 2.8 years in a GC naïve 12-year-old to a bone age delay of 8.2 years in a 16-year old who had been treated with Prednisolone from the age of 4. The results of subgroup analyses are summarised in Table 2**.**

**Table 2:** Difference in years between chronological age (CA) and bone age (BA), grouped by glucocorticoid (GC) regimen

	n	Mean CA (SD)	Mean BA (SD)	Mean diff CA-BA (95% CI of diff)
GC treated	44	10.8 (3.1)	9.1 (2.2)	1.6 (1.1, 2.1)*
GC naïve	12	8.9 (5.8)	9.0 (6.1)	−0.2 (− 0.9, 0.5)
GC previously	6	16.6 (1.4)	16.2 (1.7)	−0.4 (−1.4, 2.3)

*The difference in CA and BA is significant for the GC treated group (*p* < 0.01). SD: Standard deviation. *CI* Confidence interval

**Fig. 2:**
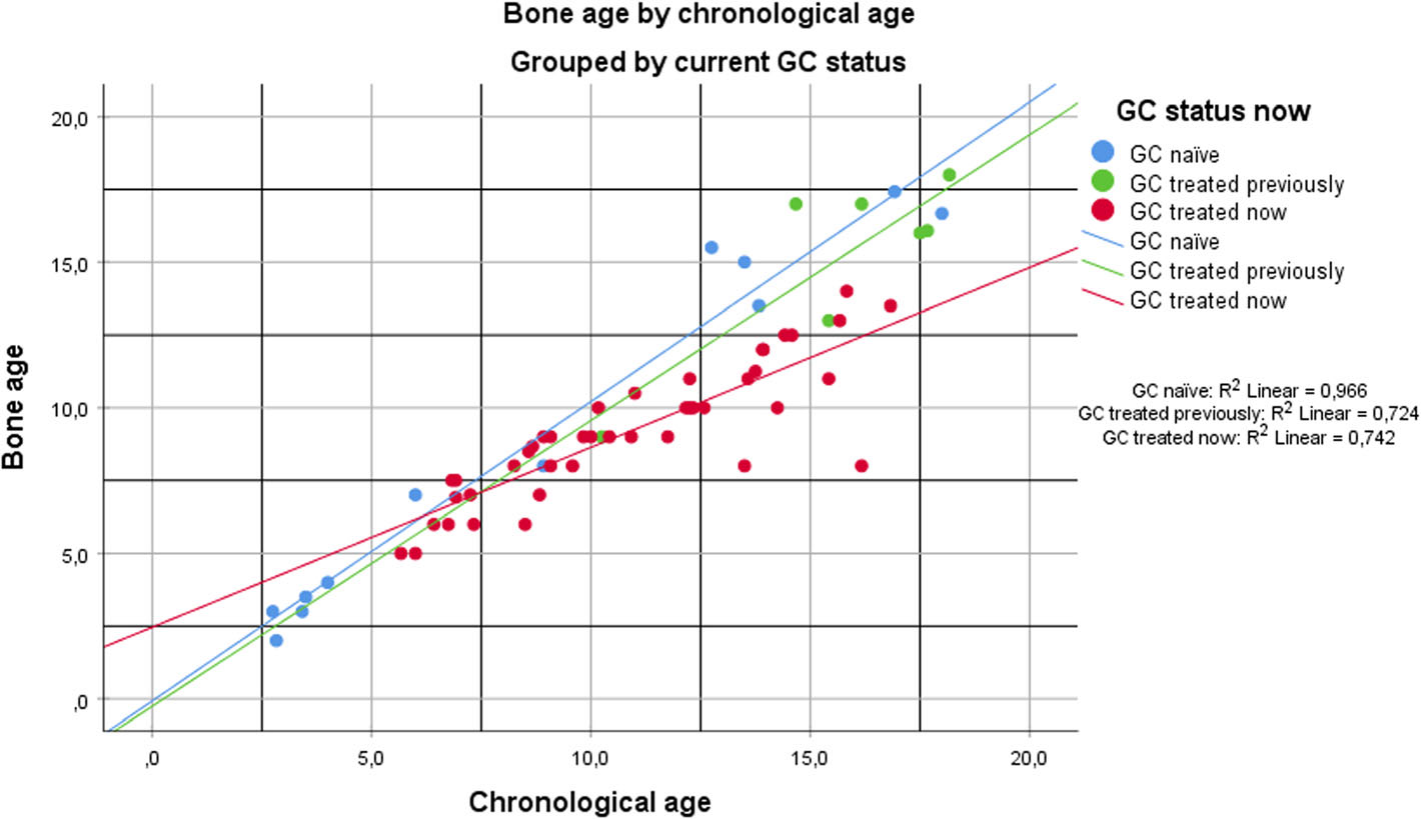
Bone age as a function of chronological age

The difference between CA and BA was significant in the GC treated group. The CA-BA difference correlated with age at evaluation (*r* = 0.6, *p* < 0.01), duration of GC treatment (*r* = 0.67, *p* < 0.01) and immature Tanner stage (*r* = 0.5, *p* < 0.01). We did not have available data to explore a possible correlation between BA delay and cumulative GC doses. An independent samples t-test revealed that any difference in skeletal delay between GC regimens (Prednisolone or Deflazacort) was not significant. Figure 2 illustrates how the skeletal maturation is retarded versus chronological age in the GC treated group and the discrepancy increases with age.

Further analyses of the GC naïve group revealed a possible trend towards bone age advancement among the older boys. However, the mean difference between chronological age and bone age in this group was not significant.

In accordance with the catch-up growth revealed on CDC growth charts of the previously GC treated boys following treatment cessation, no significant difference was found between chronological age and bone age in this group.

### Evaluation of growth corrected for delayed bone age

The mean CDC height percentiles were low for all groups (Table 1), with a statistically significant difference in mean height percentiles between GC treated and GC naïve participants.

When height percentiles were corrected for bone age delay, the number of participants with a height below the 5th CDC percentile was reduced from 31 to 13 (from 52.5 to 22.0%). Two of the 13 boys were GC naïve, 4 were prescribed Prednisolone and 7 were prescribed deflazacort.

Between-group analysis of variance based on the bone age corrected height percentiles revealed that the difference in height between GC naïve and GC treated groups was no longer significant. Thus, the stunted growth of the GC treated DMD boys was reflected in delayed skeletal maturation.

### Bone mineral density

Validated DXA scans were available for both compartments for 31/39, only L2-L4 for 2/39 or only TBLH for 6/39 participants. The total mean BMD Z-scores were − 1.89 (− 2.38, − 1.40) for L2-L4 and − 2.30 (− 2.59, − 2.00) for TBLH.

Of the 39 boys, only one was GC naïve, while three were previously GC treated. As any significant differences in BMD Z-scores between GC naïve, previously GC treated and currently GC treated boys could not be determined due to insufficient power, further statistical analyses included the GC treated subgroup only (*n* = 35).

The results are summarised in Table 3.

**Table 3:** Mean L2-L4 and TBLH chronological age based BMD Z-scores for different age groups of GC treated boys with DMD, with standard deviations (SD) and 95% Confidence Intervals for mean

CA based BMD	6–12 years	13–18 years
L2 – L4 Z-score	*n* = 18	*n* = 12
Mean (SD), 95% CI for mean	− 1.19 (1.10), − 1.73, − 0.64	− 2.94 (1.23), − 2.72, − 2.16^a^
TBLH Z-score	*n* = 23	*n* = 11
Mean (SD), 95% CI for mean	− 2.02 (0.71), − 2.33, − 1.71 ^a^	−3.03 (0.64), − 3.46, − 2.59 ^a^

^a^ BMD Z-scores <− 2 for L2 – L4 in the 13–18 years age group and for TBLH in both age groups

Mean BMD Z-scores dropped below − 2 from shortly before 12 years of age in the GC treated subgroup. Low Z-scores were significantly correlated with advanced age, long duration of GC treatment, and immature Tanner stage, but not with ambulatory status.

### Bone mineral density Z-scores corrected for delayed bone age

Validated bone age adjusted DXA scans were available for both compartments for 29/34, only L2-L4 for 3/34 or only TBLH for 2/34 participants. Again, data from only one GC naïve and two previously GC treated participants were available, and statistical analyses could only be performed on data from 31 GC treated boys. The effects of replacing chronological age with bone age are summarised in Table 4.

**Table 4:** Mean L2-L4 and TBLH bone age based BMD Z-scores for different age groups of GC treated boys with DMD, with standard deviations (SD) and 95% Confidence Intervals for mean

BA corrected BMD	6–12 years	13–18 years
L2 – L4 Z-score	*n* = 17	*n* = 12
Mean (SD), 95% CI for mean	− 1.08 (1.14), − 1.67, − 0.49	− 1.63 (1.23), − 2.40, − 0.85
TBLH Z-score	*n* = 19	*n* = 10
Mean (SD), 95% CI for mean	−1.62 (0.68), − 1.94, − 1.29	−2.34 (0.92), − 2.30, − 1.68^a^

^a^BMD Z-scores <− 2 only for TBLH in the 13–18 years age group

There was a significant correlation between the BMD Z-scores based on chronological age and the bone age adjusted parallels. As expected, Z-scores based on chronological age were negatively correlated with the CA-BA-difference, indicating that the lowest Z-scores were found in the boys with the most severe skeletal delay.

**Fig. 3:**
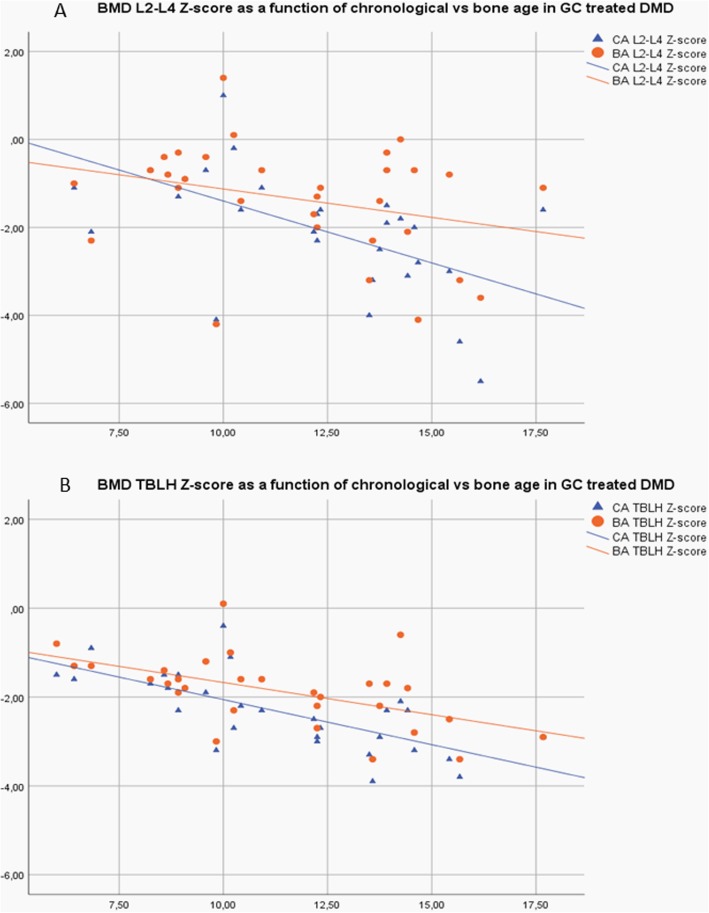
**a**: L2-L4 BMD Z-scores are significantly adjusted towards normal values (> − 2) when chronological age (CA) is replaced by bone age (BA). **b**: TBLH BMD Z-scores are significantly adjusted towards normal values (> − 2) when chronological age (CA) is replaced by bone age (BA)

The increase in BMD Z-scores for the GC treated group was significant when chronological age was corrected for skeletal delay. A paired samples T-test revealed a mean increase in BMD Z-score of 0.65 (0.40, 0.90) and 0.47 (0.31, 0.63) for L2-L4 and TBLH respectively. Fig. 3a-b illustrates the effects of age and of replacing CA with BA in the calculation of BMD Z-scores.

When chronological age was corrected for skeletal delay, BMD Z-scores were adjusted towards values within normal variation although for TBLH mean values remained below − 2 in the older age group. Thus, failing to consider skeletal delay in the evaluation of DXA BMD Z-scores led to underestimation of Z-scores.

### Appendicular and vertebral fractures

Five of 62 boys (8.1%) had sustained symptomatic vertebral fractures (VFs) confirmed by lateral radiographs, of whom three were still ambulant at the time of fracture. All five boys were among the 25 boys treated with deflazacort, while there were no confirmed VFs among 19 boys treated with Prednisolone, 12 GC naïve and 6 previously GC treated boys. All five related the VF to minor trauma (fall from own height, driving too fast over speed bump, positioning/lifting from chair). The median age was 11.5 years, while the youngest was only 6.5 years old at the time of VF.

The data revealed that all five boys had a delayed bone age of 1.8–2.3 years and BA-corrected TBLH BMD Z-scores below − 2 (− 2.8, − 3.4) at the time of our study.

Twelve of 62 boys (19.4%) had experienced long bone (LB) fractures. Four of 12 GC naïve boys (33.3%) sustained LB fractures at a young age (1.5–4.8 years), all related to falls. One of 19 Prednisolone treated boys (5.3%) sustained a femur fracture at age 7.8 years after a fall from the top of a bunk bed. Five of 25 deflazacort treated boys (20.0%) fractured an arm (2), femur (2) or clavicle (1) at the age of 3.0–9.8 years, all related to falls. Among 6 previously GC treated boys, 4 (66.7%) sustained LB fractures when they were 11.8–17.5 years old, all non-ambulant and all related to falls from chair or positioning.

## Discussion

We examined the extent of delay in skeletal maturation and the relationship between bone age delay, growth and DXA measured BMD Z-scores in GC treated boys with DMD. Our results revealed a significant delay in skeletal maturation in this patient group. The delay increased with age and duration of GC treatment, and for some boys the delay was up to several years. Our study did not reveal any significant difference in bone age delay between groups treated with prednisolone and deflazacort. The bone age of the GC naïve participants was within normal variation. Interestingly, the bone age of previously GC treated participants was comparable to GC naïve participants, indicating that the delay in skeletal maturation may be reversible upon termination of GC treatment.

The use of the G&P atlas of normal skeletal maturation of the hand and wrist [39] has several limitations [42]. The reference population of the atlas were healthy, well-nourished, middle-class, Caucasian American children and adolescents, who underwent normal pubertal development and growth spurt in the 1930s. Recent studies suggest that skeletal maturation may be reached earlier and that maturation may differ considerably between individuals and ethnicities, and with nutrition, disease and medication [42–45]. However, given its limitations, the G&P atlas still supplies valuable information about the skeletal maturation of individuals over time.

In contrast to our results, a previously published study found that bone age was compatible with chronological age in 33 GC treated boys with DMD [46]. The boys were 8.4–11.2 years old and had been treated with GCs for 32–66 months. Our participants were older and had been GC treated for a longer period, which possibly explains why bone age delay was not revealed in the previous study.

In our cohort the GC treated group was lower in height than the GC naïve group. A limitation of our study is the use of different measurement methods (standing height, lay flat or arm span), as well as possible measurement error due to lower limb contractures and lumbar lordosis. Familial short stature was not investigated, and historic growth data collected from medical records may have been inaccurate. However, both the low actual height and the increasing arrest of linear growth in GC treated DMD over time is in accordance with previous studies [13]. We found that much of the difference in height between the groups was eliminated when the height of the GC treated boys was corrected for bone age delay, and was no longer significant. Importantly, the number of boys with a height below the 5th percentile was reduced from 31 to 13 when age was corrected for skeletal delay.

In contrast to previous studies [13], the difference in weight and iso-BMI between groups was not significant. The lack of significant differences in weight in our study may however be due to insufficient power.

Previous studies have examined the effect of bone age delay on BMD Z-scores in other conditions, presenting results that correspond well with our findings [47, 48]. A study of paediatric brain tumour patients found a lack of correlation between chronological age and bone age BMD Z-scores in a subgroup of peripubertal males aged 11–15 years [47]. The authors suggested that even a small difference between bone age and chronological age greatly impacted the BMD Z-score due to the increased rate of bone mineralisation following the rapid expansion of bone volume related to normal pubertal growth spurt in boys. Thus, bone age adjustment should be added to BMD evaluations for children exhibiting abnormal growth velocities [47].

In a study of 151 healthy children and 61 children with metabolic and bone disorders, significant differences were found between BMD Z-scores based on chronological age and bone age respectively, with increased Z-scores after recalculation of BMD according to bone age in pubertal boys and children with pituitary deficiency [48]. It was suggested that considering a delayed bone age in the interpretation of BMD in the setting of pituitary hormone deficiency prevents an overestimation of skeletal deficits [19].

The clinical significance of our study with respect to the evaluation of fracture risk is yet unclear. Lateral spine radiographs of asymptomatic boys were not available, and the number of VFs may be considerably underestimated [49]. All five participants with radiologically confirmed VFs were treated with deflazacort, and the prevalence of LB fractures was higher among the deflazacort treated boys than the prednisolone treated group. However, the deflazacort treated boys were older than the prednisolone treated boys at the time of our study. Bone age adjusted BMD Z-scores at the time of VFs are not available, so that we do not have the data to explore the “true” risk of VFs in relation to corrected Z-scores. A previous study of otherwise healthy fracture-prone children found a bone-age adjusted BMD Z-score ≤ − 2.0 in only 8% of a cohort who had sustained fractures, although BMD Z-scores were significantly lower in fracture-prone children when compared to controls [50]. Interestingly, the same study found asymptomatic VFs in 15% of the fracture-prone children with a history of only appendicular fractures when screened by spinal radiography. Spinal radiographs of the controls were not available [50]. We argue that there is a need for larger, prospective studies in which bone age adjusted BMD Z-scores are reviewed in connection with lateral spine radiographs of boys with DMD on different GC regimens.

VFs can occur in children even with BMD Z-scores within normal range [4, 20, 51], and Z-scores may vary substantially depending on the normative database used [52]. The role of DXA in the evaluation of fracture risk has been altered accordingly in the 2018 DMD care considerations [51]. A low BMD Z-score is not an independent indication for bisphosphonate treatment, but rather serves as an adjuvant tool in the overall assessment of the bone health of the individual patient over time. Our results indicate that the significance of a fall in BMD Z-scores over time can only be evaluated in light of advancement or arrest in skeletal maturation over the same period of time. We suggest that if skeletal maturation is arrested, an apparent fall in BMD Z-scores may be erroneously weighted in the decision-making process regarding GC adjustments or indication for bone sparing treatment of the patient.

The results from our study correspond to a recent report of bone age delay among 12 GC treated DMD patients, as well as higher bone age adjusted BMD Z-scores for all patients [53]. Our results support the authors’ argument that the failure of DXA BMD Z-scores to predict fractures in GC treated DMD may be due to the lack of appropriate reference databases and failure to correct age-based BMD Z-scores for delay in skeletal maturation.

GC induced hypogonadotropic hypogonadism is a well-established consequence of long-term GC treatment [14]. We found delayed or absent signs of puberty among the adolescents in the GC treated group, while the GC naïve boys progressed through normal puberty. There was a relation between pubertal delay and low BMD Z-scores. As the rate of mineral accrual is linked more closely to skeletal maturation and pubertal development than to chronological age [18, 54], we suggest that testosterone replacement therapy should be considered in the presence of confirmed hypogonadism in steroid treated boys with DMD of pubertal age, not only in order to induce male secondary gender characteristics [35], but also to improve skeletal maturation and bone mineralisation as part of the pubertal development.

We found that the delay of skeletal maturation was more pronounced in the carpals than in the phalanges (Fig. 1) [25]. The rate of bone turnover in the trabecular compartment, such as the carpals and vertebrae is more rapid than that in the cortical one, and there is growing evidence that the two anatomical sites can react differently in disease states and as a result of specific treatments [9, 54, 55]. GCs have an affinity for trabecular bone [54], and failure of bone formation rather than increased bone resorption seems to be the main mechanism underlying glucocorticoid-associated bone loss [9, 10, 56]. A possible connection between marked delay of the maturation of the carpal bones and low vertebral BMD remains to be studied. If such a connection is found, the delayed carpal maturation might be used as an indication for vertebral fracture risk in GC treated DMD in the future.

In children with short stature, the two-dimensional nature of DXA will systematically underestimate bone density. Several mathematical methods of calculation have been demonstrated to improve the clinical accuracy of DXA in this situation, of which BMAD and/or HAZ are endorsed by the ISCD [19]. In a recent study, spine BMAD reference ranges are provided based on the Bone Mineral Density in Childhood Study (BMDCS) [57]. The study includes 2014 children aged 5–19 years at baseline. However, children of height, weight or BMI below the third percentile or above the 97th percentile, delayed or advanced pubertal development, use of medications known to influence bone metabolism or medical conditions that threatened normal bone accretion were excluded. The BMDCS cohort thus has important limitations in assessing children with an increased risk of fractures, such as GC treated boys with DMD [20, 58].

Further, we have demonstrated that the GC treated boys with DMD are not only short in stature, but suffer from significant delay of skeletal maturation. Calculation of BMAD or HAZ does not correct for this delay. Correcting BMD for bone age can be done directly and requires no assumptions about bone depth. We suggest that this method, which is relatively easy to perform, provides a useful impression of BMD based on the actual maturation of the bones.

Moreover, there is a need for further and larger studies of bone health and endocrine care in DMD, as well as for disease-specific reference databases for the evaluation of DXA results [19, 53, 59]. We suggest that such studies also include assessment of skeletal maturation.

## Conclusion

Our study reveals significant delay in the skeletal maturation of boys with GC treated DMD. Our results indicate that considering skeletal delay in the evaluation of growth and BMD Z-scores is imperative in the evaluation of bone health and decision-making concerning short stature and fracture risk of GC treated boys with DMD.

## Data Availability

The datasets generated and analysed during the current study are not publicly available as they contain information that could compromise research participant privacy and consent. The data are available from the corresponding author (EA) on reasonable request.

## Abbreviations

BA: Bone age
BMAD: Bone mineral apparent density
BMC: Bone mineral content
BMD: Bone Mineral Density
BMI: Body mass index
CA: Chronological age
CDC: Centers for Disease Control and Prevention
DMD: Duchenne muscular dystrophy
DXA: Dual-energy X-ray absorptiometry
G&P: Greulich and Pyle
GC: Glucocorticoid
HAZ: Height-for-age Z-score
ISCD: International Society for Clinical Densitometry
LB: Long bone
SD: Standard deviation
TBLH: Total body less head
VF: Vertebral fracture
